# Flow-Mediated Dilatation in the Assessment of Coronary Heart Disease: A Meta-Analysis

**DOI:** 10.1155/2022/7967324

**Published:** 2022-09-28

**Authors:** Xiaoyong Xiao, Xiang Li, Xiaohua Xiao, Jingjing Wang, Dehong Liu, Zhe Deng

**Affiliations:** ^1^Department of Emergency, Shenzhen Second People's Hospital, The First Affiliated Hospital of Shenzhen University, Shenzhen 518035, China; ^2^Department of Ultrasound, Shenzhen Pingle Orthopaedic Hospital, Shenzhen 518000, China; ^3^Department of Geriatric Medicine, Shenzhen Second People's Hospital, The First Affiliated Hospital of Shenzhen University, Shenzhen 518035, China

## Abstract

Endothelial dysfunction may contribute to the increased morbidity and mortality associated with coronary heart disease (CHD). Flow-mediated dilatation (FMD) is the most popular noninvasive method for vascular endothelial function evaluation. This meta-analysis aimed to investigate the association between FMD and CHD. We searched the publications listed in the PubMed, Web of Science, Scopus, and Embase databases. Stata 14 software was used to analyze the data. Standardized mean difference (SMD) was used to calculate FMD levels, and the effect sizes were expressed with a 95% confidence interval (CI). I^2^ statistics were used to evaluate statistical heterogeneity. In this meta-analysis, 9 studies enrolled a total number of 943 participants, including 534 (56.63%) patients with CHD and 409 controls (43.37%). We found that patients with CHD showed a significantly lower FMD than the controls (SMD −0.706%; 95% CI: −0.985, −0.427; *P*=0.001) with high heterogeneity. In addition, funnel plot analysis suggested asymmetry that could be evidence of publication bias. But sensitivity analyses show that there were no influential studies. This meta-analysis provides evidence that patients with CHD show a significantly lower FMD than controls and highlights the literature on FMD as a hallmark in CHD diseases.

## 1. Introduction

The vascular endothelium plays an essential role in various pathological and physiological processes, for instance, regulating vascular homeostasis, vasoconstriction and dilation, thrombosis, fibrinolysis, inhibition of inflammation, and smooth muscle cell function [1]. An imbalance between the magnitude of injury and the repair capacity of the endothelium is a key factor in the occurrence and development of various cardiovascular diseases, such as myocardial infarction, stroke, and hypertension [2]. Accumulating evidence has confirmed that endothelial dysfunction is considered an important pathological feature of early atherosclerosis [3]. Endothelial function deteriorates during the natural history of cardiovascular diseases, suggesting that it may be a potential biomarker in the context of this disease and warrants further investigation [4]. Therefore, early detection of vascular endothelial dysfunction, timely intervention, and guided treatment are significant for maintaining cardiovascular health and reducing morbidity and mortality associated with cardiovascular diseases and medical costs [5].

Currently, invasive and noninvasive methods are commonly used to evaluate vascular function. Invasive tests require the injection of acetylcholine into human coronary arteries, but their clinical application is limited because they are invasive, time-consuming, and expensive, and they are not recommended for healthy or asymptomatic patients [5]. The noninvasive endothelial function detection method first appeared in the 1990s. With the continuous development of basic and clinical research, the medical industry has paid increasing attention to examining endothelial function in cardiovascular diseases, and noninvasive vascular endothelial function detection has become a research hotspot [6]. At present, brachial artery flow-mediated dilatation (FMD) is the most popular method for evaluating vascular endothelial function evaluation [7]. It has the advantages of less or no trauma, relatively low cost, simple operation, good repeatability, and wide application and is more acceptable [7]. Therefore, FMD detection has been widely adopted in cardiovascular research in clinical trials.

Many large prospective cohort studies have accepted FMD as an adjunctive marker of coronary heart disease (CHD). Several studies found that a decreased FMD could increase the risk of CHD [8, 9]. However, some of these studies have been summarized in qualitative reviews. Therefore, we conducted a meta-analysis that aims to describe FMD as a potential biomarker for CHD.

## 2. Methods

### 2.1. Search Strategy

This meta-analysis followed the guidelines of the preferred reporting items. We searched the publications listed in the electronic databases MEDLINE (source PubMed, January 1, 2001, to January 1, 2021) and Embase (January 1, 2001, to January 1, 2021) using the following text and keywords in combination both as MeSH terms and text word: “flow-mediated dilatation” and ‘‘coronary artery disease” or “coronary heart disease” or “coronary vascular disease.”

### 2.2. Study Inclusion/Exclusion Criteria

Inclusion criteria of this study were as follows: patients with coronary heart disease; FMD brachial artery in CHD and control was detected by high-resolution ultrasound methods, and the brachial artery FMD was calculated as [(reactive hyperemia diameter of the brachial artery-baseline diameter)/baseline diameter × 100%]; and the enrolled studies were prospectively or retrospectively designed. Exclusion criteria were as follows: patients with infections, tumors, and immune disease; incomplete FMD data; literature that was duplicated and poorly designed; and reviews.

### 2.3. Search and Screening

Two investigators independently screened the titles and abstracts of the studies and excluded those that did not meet the inclusion criteria. After screening the full text, the studies that met the requirements were selected according to the inclusion and exclusion criteria. If two authors had objections to the extracted literature, a third investigator was invited to arrive at the final conclusion. Finally, the articles were written according to the systematic reviews and meta-analyses guidelines.

### 2.4. Data Analysis and Statistical Methods

Stata 14 software was used to analyze the data. Standardized mean difference (SMD) was used to calculate FMD levels, and the effect sizes were expressed with a 95% confidence interval (CI). Chi-square Cochran's *Q* test and the I^2^ statistic were used to assess the heterogeneity between studies. In detail, if there was no heterogeneity (I^2^ <50%, *P* > 0.05), the fixed-effects model was used for analysis. If heterogeneity existed (I^2^ ≥ 50%, *P* ≤ 0.05), the random-effects model was used for analysis. Sensitivity analysis was conducted to determine the stability and credibility of the results. Funnel plots and shear complement methods were used to detect publication biases.

## 3. Results

### 3.1. Literature Results

**Figure 1:**
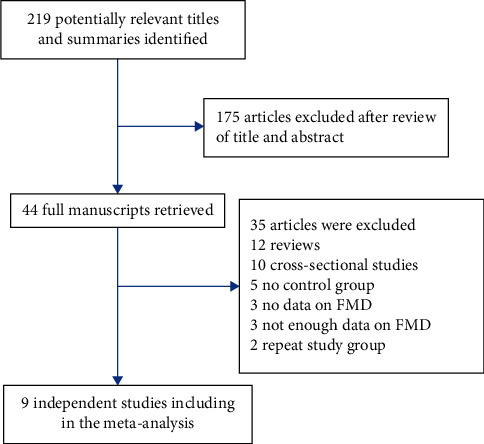
Flowchart of the selection of studies included in meta-analysis.

The results of the literature search are shown in Figure 1. After reviewing the titles and abstracts, we retrieved 219 unique citations and eliminated 175. Of the 44 full manuscripts retrieved, 35 were excluded because 12 were reviews, 10 were cross-sectional studies, 5 had no controls, 3 had no data on FMD, 3 did not have enough data on FMD, and 2 were repeat studies. Ultimately, 9 articles were included in the meta-analysis [10–18].

### 3.2. Study Characteristics

All included studies were observational and were prospectively designed. As shown in Table 1, 9 studies used ultrasound-based FMD to determine the endothelial function in this meta-analysis. A total of 943 participants were included: 534 patients with coronary heart disease (56.63%) and 409 controls (43.37%). Most controls were healthy. The study population is mainly European and Asian. The mean age for the entire study population was 58 years, and males represent 75.59% of them. Most patients in the control group were healthy (Table 1).

### 3.3. Endothelial Function Assessment in Cardiovascular Diseases

**Figure 2:**
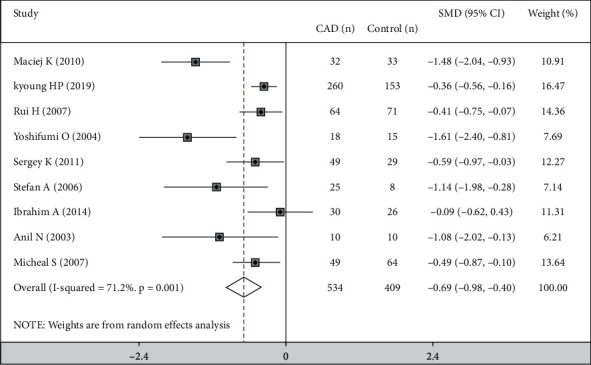
SMD with 95 % CI of FMD in CHD and control.

In 9 studies, heterogeneity was I^2^ = 71.2% and *P*=0.001, suggesting heterogeneity was statistically significant among these studies. Therefore, a random-effects model was used to analyze the data in this meta-analysis. As shown in Figure 2, we found that the 534 coronary heart disease patients showed a significantly lower FMD than the 409 controls (MD −0.690%; 95% CI: −0.981, −0.398; *P*=0.001), suggesting that endothelial dysfunction correlates with coronary heart diseases.

### 3.4. Publication Bias

**Figure 3:**
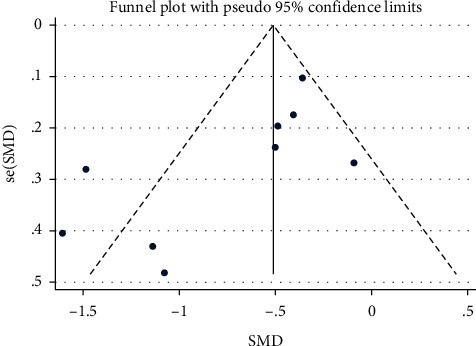
Funnel plot for analyzing publication bias.

**Figure 4:**
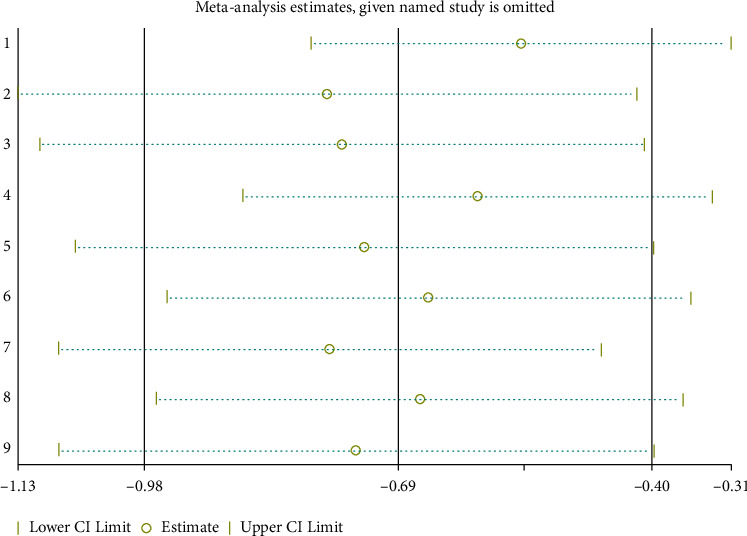
Sensitivity analysis of the meta-analysis.

Publication bias was assessed using a funnel plot analysis. As shown in Figure 3, funnel plot analysis confirmed the existence of significant publication bias, suggesting that publication bias was present in this meta-analysis. Thus, we used the trim and fill method to correct the funnel asymmetry caused by publication bias, and we obtained a similar SMD, indicating that although there was a certain publication bias, the results were stable and reliable. As shown in Figure 4, a sensitivity analysis was performed by culling the included studies one by one, and the SMD was reweighed to evaluate the stability of the results. Similarly, there was a significant difference in FMD between patients with CHD and control, indicating stable results.

## 4. Discussion

Endothelial cell damage and dysfunction play an important role in developing the process [3]. Studies have shown that arteriosclerosis is related to CHD and can increase the risk of cardiovascular disease [19, 20]. In recent clinical studies, FMD, an accepted method for noninvasive assessment of systemic endothelial function, has been extensively examined [21]. Accumulating evidence indicates that FMD is a hallmark of CHD [10–18]. However, few reviews have summarized the relationship between endothelial function and CHDs. In this study, we systematically reviewed the scientific literature and assessed the differences in endothelial function between the CHD and control groups.

The present meta-analysis results consistently showed that CHDs might be associated with endothelial dysfunction, as evaluated by ultrasound-based FMD of the brachial artery. In particular, we demonstrated that 534 patients with CHD showed a significantly lower FMD than the 409 controls (SMD −0.690%; 95% CI: −0.981, −0.398; *P*=0.001), indicating that patients with CHD show a reduced vasodilatory response and endothelial function. Therefore, evaluation of FMD can be a useful noninvasive diagnostic tool for CHD risk assessment.

Many cardiovascular risk factors affect endothelial function, such as race, age, estrogen, hypertension, diabetes, and smoking [22]. Although most patients with CHD have a history of smoking, the relationship between FMD and CHD appears to be more complex, and smoking habits may not fully explain the decline in FMD of CHD in this clinical setting.

The funnel plot analysis confirmed the existence of significant publication bias. However, we obtained a similar SMD by the trim and fill method, and there was no obvious difference compared with previous results, possibly because of low power due to the small number of studies [23]. In addition, we obtained the stability of the results in this meta-analysis, which was evaluated by sensitivity analysis. Therefore, we conclude that the results were stable and reliable.

Accumulating evidence indicates that early endothelial dysfunction is a reversible disorder, and several interventions, such as exercise and bilirubin or other medicine, can increase endothelial function and reduce CVD risk [24, 25]. If FMD is taken to be an early marker for CHD, vascular endothelial dysfunction will be evaluated early. Timely intervention and guided treatment can reverse endothelial dysfunction and reduce the morbidity and mortality associated with CHD.

Some potential limitations of our study must be discussed. First, there was a publication bias in this meta-analysis. However, the combined SMD was consistent with the uncut and complemented conclusions, indicating that although there was a certain publication bias, the results were stable and reliable. Second, heterogeneity among the studies was generally significant, and it was not possible to conclusively ascertain sources of heterogeneity. Finally, the small number of studies and participants limited the accuracy of the results. Further efforts will be made to conduct further studies in the future.

## 5. Conclusion

In conclusion, this meta-analysis provides evidence that patients with CHD show a significantly lower FMD than controls and highlights the literature on FMD as a hallmark in CHD diseases. Although limited in number, these studies provide important evidence that FMD should be considered a worthy biomarker for assessment in future research.

## Figures and Tables

**Table 1:** Clinical data of patients with CHD and controls in included studies.

Study	Country	CHD	Control	Pop (*n*)	Man (%)	Age (mean) (years)		Hypertension (%)		Diabetes (%)		CAD (%)		Smoking (%)	
						CHD	Control	CHD	Control	CHD	Control	CHD	Control	CHD	Control
Maciej K (10)	Poland	CAD	No CAD	65	0	50.3	50.2	66	61	44	15	25	24	75	39
Kyoung HP (11)	Korea	CAD	No CAD	413	76.5	59.4	58.1	56.9	52.9	26.9	22.2	13.8	15	37.7	25.5
Rui H (12)	China	CAD	Healthy	135	70.3	62.4	54.9	62.5	34.8	20.3	8.7	NA	NA	37.3	20.2
Yoshifumi O (13)	Japan	CAD	Healthy	33	NA	61.3	64.7	NA	NA	NA	NA	NA	NA	56	40
Sergey K (14)	Russia	CAD	Healthy	78	100	43	43	4	17	NA	NA	80	NA	86	52
Stefan A (15)	Sweden	CAD	Healthy	33	NA	57	53	NA	NA	NA	NA	NA	NA	48	12
Ibrahim A (16)	Turkey	CAD	Healthy	56	100	52.5	49.69	NA	NA	36	0	NA	NA	77	86
Anil N (17)	Canada	CAD	Healthy	20	100	56	38	10	0	20	0	NA	NA	30	0
Micheal S (18)	Israel	CAD	Healthy	110	83	60	55	48	36	24	6	NA	NA	17	14

CHD: coronary heart disease. CAD: coronary artery disease.

## Data Availability

The data used to support the findings of this study are available from the corresponding author or the first author upon request.
